# Small molecule inhibition of 6-phosphofructo-2-kinase suppresses t cell activation

**DOI:** 10.1186/1479-5876-10-95

**Published:** 2012-05-16

**Authors:** Sucheta Telang, Brian F Clem, Alden C Klarer, Amy L Clem, John O Trent, Richard Bucala, Jason Chesney

**Affiliations:** 1 James Graham Brown Cancer Center, Division of Medical Oncology and Hematology, Department of Medicine, University of Louisville, Louisville, KY, USA; 2 Division of Rheumatology, Department of Medicine, Yale University School of Medicine, New Haven, CT, USA; 3 James Graham Brown Cancer Center, University of Louisville, 505 South Hancock Street, #424, Louisville, KY, 40202, USA; 4 University of Louisville, 505 South Hancock Street, #423, Louisville, KY, 40202, USA

**Keywords:** Glycolysis, 6-Phosphofructo-2-kinase/fructose-2, 6-bisphosphatase, Fructose-2, 6-bisphosphate, T cell

## Abstract

**Background:**

T cell activation is associated with a rapid increase in intracellular fructose-2,6-bisphosphate (F2,6BP), an allosteric activator of the glycolytic enzyme, 6-phosphofructo-1-kinase. The steady state concentration of F2,6BP in T cells is dependent on the expression of the bifunctional 6-phosphofructo-2-kinase/fructose-2,6-bisphosphatases (PFKFB1-4) and the fructose-2,6-bisphosphatase, TIGAR. Of the PFKFB family of enzymes, PFKFB3 has the highest kinase:bisphosphatase ratio and has been demonstrated to be required for T cell proliferation. A small molecule antagonist of PFKFB3, 3-(3-pyridinyl)-1-(4-pyridinyl)-2-propen-1-one (3PO), recently has been shown to reduce F2,6BP synthesis, glucose uptake and proliferation in transformed cells. We hypothesized that the induction of PFKFB3 expression may be required for the stimulation of glycolysis in T cells and that exposure to the PFKFB3 antagonist, 3PO, would suppress T cell activation.

**Methods:**

We examined PFKFB1-4 and TIGAR expression and F2,6BP concentration in purified CD3^+^ T cells stimulated with microbead-conjugated agonist antibodies specific for CD3 and the co-stimulatory receptor, CD28. We then determined the effect of 3PO on anti-CD3/anti-CD28-induced T cell activation, F2,6BP synthesis, 2-[1-^14^C]-deoxy-d-glucose uptake, lactate secretion, TNF-α secretion and proliferation. Finally, we examined the effect of 3PO administration on the development of delayed type hypersensitivity to methylated BSA and on imiquimod-induced psoriasis in mice.

**Results:**

We found that purified human CD3^+^ T cells express PFKFB2, PFKFB3, PFKFB4 and TIGAR, and that anti-CD3/anti-CD28 conjugated microbeads stimulated a >20-fold increase in F2,6BP with a coincident increase in protein expression of the PFKFB3 family member and a decrease in TIGAR protein expression. We then found that exposure to the PFKFB3 small molecule antagonist, 3PO (1–10 μM), markedly attenuated the stimulation of F2,6BP synthesis, 2-[1-^14^C]-deoxy-D-glucose uptake, lactate secretion, TNF-α secretion and T cell aggregation and proliferation. We examined the *in vivo* effect of 3PO on the development of delayed type hypersensitivity to methylated BSA and on imiquimod-induced psoriasis in mice and found that 3PO suppressed the development of both T cell-dependent models of immunity *in vivo*.

**Conclusions:**

Our data demonstrate that inhibition of the PFKFB3 kinase activity attenuates the activation of T cells *in vitro* and suppresses T cell dependent immunity *in vivo* and indicate that small molecule antagonists of PFKFB3 may prove effective as T cell immunosuppressive agents.

## Background

Fifty years ago, the activation of T cells with mitogens such as phytohaemagglutinin was observed to stimulate glucose uptake and conversion into lactate [1-4]. Although diverting the flux of carbons away from the tricarboxylic acid cycle and into lactate reduces the net ATP production from glucose, the flow of glycolytic intermediates provides a ready supply of anabolic precursors needed for cytokine production and cell proliferation. The stimulation of glycolysis observed in activated T cells is believed to be mediated by an increase in the expression of the Glut1 glucose transporter and several glycolytic enzymes [5,6], and by an increase in the concentration of fructose-2,6-bisphosphate (F2,6BP), an allosteric activator of 6-phosphofructo-1-kinase (PFK-1) [7]. Importantly, a potential requirement of F2,6BP for the stimulation of glycolysis in T cells was suggested by the observation that the immunosuppressive steroid, triamcinolone acetonide, caused a marked decrease of F2,6BP and ATP in rat thymocytes [8]. However, no studies to date have demonstrated the obligatory requirement of F2,6BP for the activation of T cells or the potential utility of targeting the production of this allosteric effector as an immunosuppressive strategy.

The steady-state concentration of F2,6BP is determined by the homodimeric bifunctional 6-phosphofructo-2-kinase/fructose-2,6-bisphosphatases (PFKFBs) which can phosphorylate fructose-6-phosphate (F6P) to F2,6BP and dephosphorylate F2,6BP to F6P, respectively [9-11], and by a monofunctional fructose-2,6-bisphosphatase, termed p53-inducible regulator of glycolysis and apoptosis (TIGAR) [12]. The PFKFB kinase domain is structurally related to the mononucleotide-binding protein family, which includes adenylate kinase, Ras and EF-Tu proteins, while the PFKFB bisphosphatase domain belongs to the phosphoglycerate mutase family [13]. Four PFKFB family members have been identified that are encoded by separate genes (*PFKFB1-4)*, expressed in different tissues, and characterized by distinct kinase:phosphatase ratios [10,14,15]. Whereas the PFKFB1 family member is predominantly expressed in the liver and skeletal muscle, PFKFB2, 3 and 4 are variably co-expressed in hematopoietic, epithelial and transformed cells [16-19]. The PFKFB3 family member has the highest kinase:bisphosphatase ratio (740:1) (20), increases F2,6BP when ectopically expressed in transformed cells (21) and, when silenced with shRNA, can reduce the proliferation and lactate secretion of anti-CD3 stimulated human T cells (22). Although homozygous genomic deletion of PFKFB3 is embryonic lethal, heterozygous genomic deletion of PFKFB3 does not cause a reduction in litter size, birth weight, development or aging despite a 50% reduction in PFKFB3 protein expression in all examined cell types [20]**,** suggesting that therapies that inhibit PFKFB3 may be well tolerated. A small molecule antagonist of PFKFB3, 3-(3-pyridinyl)-1-(4-pyridinyl)-2-propen-1-one (3PO), was recently found to reduce the F2,6BP, glucose uptake and proliferation of transformed cells and to suppress the growth of xenograft tumors *in vivo*[21]. Importantly, 3PO and its optimized derivatives do not affect serum glucose, red blood cell or white blood cell concentration when administered daily to mice and rats (*unpublished observations)*. Based on these data, we hypothesized that the stimulation of glycolysis in T cells may be dependent on the induction of PFKFB3 activity and that exposure to 3PO would suppress the activation of T cells without causing significant toxicity.

We report that the engagement of the T cell receptor (TCR) and co-stimulatory receptor, CD28, causes an increase in PFKFB3 protein expression and a decrease in TIGAR expression that is coincident with an increase in the steady-state concentration of F2,6BP. We also find that 3PO markedly reduces anti-CD3/anti-CD28-induced F2,6BP synthesis, glucose uptake, lactate secretion, TNF-α secretion and proliferation. Finally, using two mouse models of immunologic responses that are dependent on T cell activation, we demonstrate that 3PO displays significant immunosuppressive activity *in vivo*. Potent PFKFB3 antagonists, including 2^nd^ and 3^rd^ generation 3PO derivatives, are currently in advanced pre-clinical development and may prove to be effective as immunosuppressive agents.

## Methods

### T cell isolation and stimulation with anti-CD3/anti-CD28-conjugated microbeads

Human mononuclear cells were separated by centrifugation through a Ficoll-Paque density gradient (GE Healthcare, Uppsala, Sweden), CD3^+^ T cells were isolated by negative selection using T cell enrichment columns (R & D Systems, Minneapolis, MN) and >98% purity was confirmed by flow cytometry. T cells then were plated in RPMI 1640 (Invitrogen, Carlsbad, CA) containing 10% fetal bovine serum (Hyclone, Logan, UT) and gentamicin 50 μg/ml at a density of 1 x 10^6^ cells/ml per well with or without anti-CD3/anti-CD28-conjugated microbeads (25 μl microbeads/ml; Dynal/Invitrogen, Oslo, Norway). For the proliferation experiments, the media were supplemented with human IL-2 (30 units/ml) (Gibco, Invitrogen, Camarillo, CA).

### Real time PCR analyses

PFKFB1-4 and TIGAR mRNA expression were determined using real-time RT**-**PCR with TaqMan probes for human PFKFB1-4, TIGAR and β-actin (Applied Biosystems, Foster City, CA) in triplicate in 96-well optical plates (MicroAMP®, Applied Biosystems). Analysis of results and fold differences between samples were determined using StepOne software (version2.1) (Applied Biosystems) and the comparative CT method. Fold change (from 0 hrs) was calculated from the ΔΔCT values with the formula (2^-ΔΔCT^) and the data are represented as the mean ± SD from triplicate measurements from three independent experiments. Statistical significance was assessed by the two-sample *t* test (independent variable).

### Protein extraction and Western blotting

Cells were harvested, washed once in PBS and solubilized in lysis buffer (Pierce Biotechnology, Rockford, IL) containing protease inhibitors. Protein samples were resolved on a 4-20% gradient SDS-PAGE gel and transferred to a PVDF membrane. After blocking in TBS-Tween 20 (0.1%) containing 5% milk, membranes were probed with anti-PFKFB3 and anti-PFKFB2 (both from Proteintech, Chicago, IL), anti-PFKFB4 (Epitomics, Burlingame, CA), anti-TIGAR (Abcam, Cambridge, MA), anti-CD69 (Novus Biologicals, Littleton, CO) or anti-β-actin (Sigma, St. Louis, MO) in TBS-Tween 20 (containing 2.5% milk). Secondary antibodies used were goat anti-rabbit or anti-mouse HRP conjugated (1:5000, Pierce Biotechnology). All Western blotting experiments were repeated for a total of 3 experiments. Scanned images were quantified by densitometric analyses using Image J software based analysis (http://rsb.info.nih.gov/ij/). Values obtained were normalized to β-actin (as a control) and expressed in densitometric units as a percentage of 0 hour expression. The data represented are the mean ± SD from triplicate measurements from three independent experiments. Statistical significance was assessed by the two-sample *t* test (independent variable).

### Exposure of human CD3^+^ T cells to 3PO

Vehicle (dimethyl sulfoxide [DMSO]; 0.1%) or 3PO at concentrations of 1, 5 or 10 μM were added to media after the addition of CD3^+^ T cells to the anti-CD3/anti-CD28-conjugated microbeads and then harvested after 0, 5, 10, 24, 48 or 72 hours for measurement of F2,6BP, 2-[1-^14^ C]-deoxy-D-glucose, ATP, and direct cellular enumeration with a New Brunswick NucleoCounter (viable T cell counts were determined as the difference between the number of unlysed T cells that were detected after staining with propidium iodide and the total number of T cells that were detected after lysis).

### F2,6BP measurements

Cells were triturated, washed twice with PBS, dissolved in 0.1 M NaOH and F2,6BP content measured using a coupled enzyme reaction following the method of Van Schaftingen *et al.*[22]. The F2,6BP concentration was normalized to total cellular protein measured by the bicinchoninic acid assay (Pierce Biotechnology). All data are expressed as the mean ± SD of three experiments. Statistical significance was assessed by the two-sample *t* test (independent variable).

### ATP measurements

Cell pellets were lysed using Passive Lysis buffer (1X, Molecular Probes, Invitrogen, Carlsbad, CA). Lysates were flash frozen (to −80°C) and thawed (to 37°C) once to accomplish complete lysis and then centrifuged (at 4°C) for 30 seconds to clear the lysates. Intracellular ATP levels were determined using a bioluminescence assay (Molecular Probes) utilizing recombinant firefly luciferase and its substrate, D-luciferin and following manufacturer’s instructions. The luminescence was read in a TD-20/20 luminometer (Turner Designs, Sunnyvale, CA) at 560 nm. The ATP values were calculated using an ATP standard curve. The protein concentrations of the lysates were estimated using the bicinchoninic acid assay (Pierce Biotechnology) and ATP was expressed as pmol per mg protein. All data are expressed as the mean ± SD of three experiments. Statistical significance was assessed by the two-sample *t* test (independent variable).

### Lactate measurements

Lactate concentrations in the media were measured using a lactate oxidase-based assay read at 540 nm (Trinity, Wicklow, Ireland). In certain experiments, lactate data were normalized to viable cell number. Experiments were repeated four times and are expressed as mean ± SD. Statistical significance was assessed by the unpaired two-tail *t*-test.

### 2-[1-^14^ C]-deoxy-D-glucose uptake

Cells were placed in glucose-free RPMI 1640 for 30 minutes, 2-[1-^14^ C]-deoxy-D-glucose (0.25 μCi/mL; Perkin Elmer, Boston, MA) was added for an additional 60 min and the cells then were washed thrice with ice-cold RPMI 1640 containing no glucose. Cell lysates were collected in 500 μL of 0.1% SDS, and scintillation counts (counts/min) were measured on 400 μL of lysate. Counts were normalized to protein concentration, and data are represented as mean ± SD from triplicate measurements from three independent experiments. Statistical significance was assessed by the two-sample *t* test (independent variable).

### TNF-α ELISA

Supernatants from T cells exposed to DMSO or 1, 5 or 10 μM 3PO for 5–24 hours were harvested and TNF-α concentration was determined using an ELISA assay (Quantikine, R & D Systems, Minneapolis, MN). The assay was performed in quadruplicate, the [TNF-α] was normalized to cell number and the data were represented as mean ± SD from three independent experiments. Statistical significance was assessed by the two-sample *t* test (independent variable).

### Delayed type hypersensitivity (DTH) model

Six BALB/c mice per group were injected subcutaneously with 250 μg methylated BSA (mBSA) (Sigma-Aldrich) at two sites in the abdomen in a combined total volume of 100 μl in a 1:1 emulsion of CFA (BD Biosciences, San Diego, CA) and saline as previously described [23]. Seven days following immunization, the mice were challenged with an injection of 50 μl of 0.5 mg/ml mBSA in saline in one rear foot pad and 50 μl saline in the other rear footpad and then administered either 3PO (0.07 mg/gm) or DMSO intraperitoneally (i.p.) 30 minutes and 12 hrs after the challenge. Footpad thickness was measured 24 hours following mBSA challenge with microcalipers. After euthanasia, popliteal lymph nodes were removed and their size was measured and total numbers of lymphocytes counted using light microscopy.

### Imiquimod-induced psoriasis

Twenty gram BALB/c mice were administered a daily topical dose of 62.5 mg of commercially available imiquimod cream (5%) (Perrigo, Allegan, MI) over a shaved area on the back for 5 days. Thirty minutes following administration, the mice were injected intraperitoneally with either DMSO or 3PO 0.07 mg/gm daily. After 5 days, the mice were photographed and then euthanized and skin and spleens resected for analyses. The data represented are the mean ± SD from three independent experiments (n = 3 mice/group/experiment).

### Histology and immunohistochemistry

Five μm sections of formalin fixed and paraffin embedded skin and spleen sections were treated with xylene to remove paraffin and rehydrated. Hematoxylin & eosin staining was performed using standard procedures. For immunohistochemical staining for CD3, deparaffinized and rehydrated sections were blocked by incubation with serum blocking buffer for 30 min at room temperature then incubated with a rabbit polyclonal antibody to CD3 (1:200, Abcam) followed by goat anti-rabbit secondary antibody for 30 min (1:500, Vector Laboratories, Burlingame, CA) and developed with alkaline phosphatase. After counterstaining with Mayer’s hematoxylin (Sigma), the sections were dehydrated, and coverslips were attached with Permount (Fisher Scientific, Pittsburgh, PA). Appropriate negative controls were used.

## Results

### Induction of PFKFB3 and TIGAR expression by human T cells after exposure to anti-CD3/anti-CD28-conjugated microbeads

**Figure 1:**
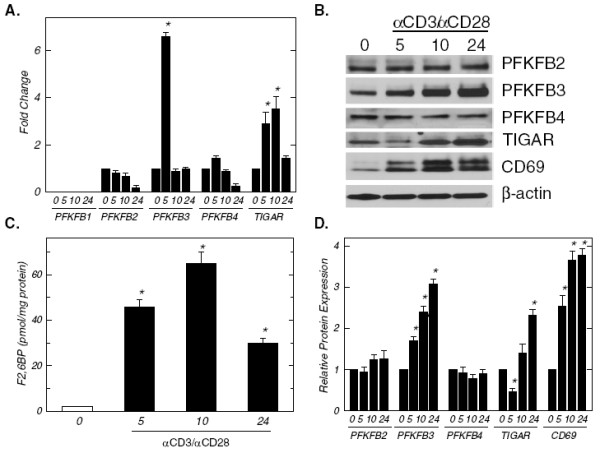
**Activation of human CD3**^**+**^**T cells increases PFKFB3 expression and intracellular F2,6BP.** CD3^+^ T cells were isolated by negative selection and then plated at a final concentration of 1 x 10^6^ cells/ml in the absence or presence of anti-CD3/anti-CD28-conjugated microbeads for 5, 10 and 24 hours. (**A**), PFKFB1-4 and TIGAR mRNA expression was determined using real-time RT-PCR analyses. (**B**), PFKFB2-4, TIGAR, CD69 and β-actin protein expression was determined by Western blot analyses. (**C**), Intracellular F2,6BP concentration was determined using a coupled enzyme assay. (**D**), Densitometric analysis of PFKFB2-4, TIGAR, CD69 and β-actin protein expression. Data are representative of three independent experiments. *p < 0.05 (relative to expression or concentration at 0 hours).

In order to assess the potential role of F2,6BP in early T cell activation, we cultured human CD3^+^ T cells in the presence of microbeads that were pre-coated with antibodies to the T cell receptor subunit, CD3, and the co-stimulatory surface ligand, CD28, which is a combination that closely mimics the signals that antigen presenting cells transmit. Initially, we examined the mRNA and protein expression of all five enzymes known to affect the intracellular concentration of F2,6BP including the four PFKFB family members and TIGAR. Purified human CD3^+^ T cells co-expressed PFKFB2, PFKFB3, PFKFB4 and TIGAR mRNA and, within five hours of activation, PFKFB3 mRNA expression increased simultaneously with a more modest but sustained increase in TIGAR mRNA expression (Figure 1A). The induction of PFKFB3 and TIGAR mRNA expression was followed by an increase in PFKFB3 protein expression but an initial decrease and then an increase in TIGAR expression that was coincident with the induction of the T cell early activation glycoprotein CD69 (Figure 1B and 1D). Since PFKFB3, which lacks significant bisphosphatase activity and TIGAR, which does not have the kinase domain, are considered to have opposing effects on the intracellular concentration of F2,6BP, we then measured the intracellular concentration of F2,6BP. We found that the F2,6BP concentration markedly increased within five hours of incubation of T cells with the anti-CD3/anti-CD28-conjugated microbeads (Figure 1C). These data suggest that the initial decrease in TIGAR expression coupled to a sustained increase in PFKFB3 expression may together contribute to the observed rise in intracellular F2,6BP during T cell activation.

### The PFKFB3 small molecule antagonist 3PO reduces anti-CD3/anti-CD28-induced F2,6BP, 2-[1-^14^ C]-deoxy-D-glucose uptake, lactate and TNF-α secretion by T cells

**Figure 2:**
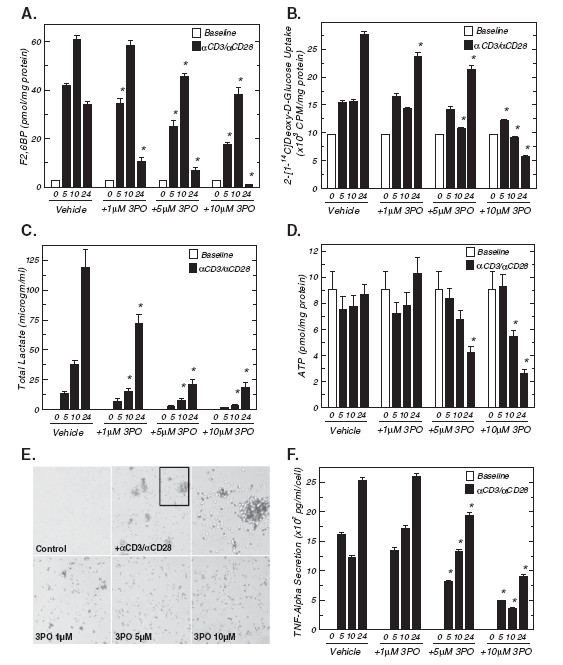
**A PFKFB3 small molecule antagonist inhibits stimulation of F2,6BP, glucose uptake, lactate secretion, ATP and TNF-α secretion caused by 10 hours of exposure to anti-CD3/anti-CD28-conjugated microbeads.** (**A**), Either vehicle (DMSO) or 1–10 μM 3PO was added to human CD3+ T cells stimulated with anti-CD3/anti-CD28-conjugated microbeads for 5, 10 and 24 hours and F2,6BP was determined. (**B**), 2-[1-^14^ C]-deoxy-D-glucose uptake after addition of anti-CD3/anti-CD28-conjugated microbeads in the presence or absence of the indicated concentrations of 3PO for 5, 10 and 24 hours. (**C**), Lactate secretion after addition of anti-CD3/anti-CD28-conjugated microbeads in the presence or absence of the indicated concentrations of 3PO for 5, 10 and 24 hours. (**D**), Intracellular ATP after addition of anti-CD3/anti-CD28-conjugated microbeads in the presence or absence of the indicated concentrations of 3PO for 5, 10 and 24 hours. (**E**), Either vehicle (DMSO) or 1–10 μM 3PO was added to human CD3^+^ T cells stimulated with anti-CD3/anti-CD28-conjugated microbeads for 10 hours and cell aggregates were assessed using light microscopy. (**F**), Secreted TNF-α after addition of anti-CD3/anti-CD28-conjugated microbeads in the presence or absence of the indicated concentrations of 3PO for 5, 10 and 24 hours was determined by ELISA. Data are representative of three independent experiments. *p < 0.05 (relative to vehicle control).

Given that stimulation of T cells causes such a large increase in the intracellular concentration of F2,6BP, we speculated that small molecule inhibition of the kinase activity of PFKFB3 would attenuate T cell activation. Initially, we examined the effect of the PFKFB3 inhibitor 3PO (1–10 μM) on the increase in F2,6BP caused by CD3/CD28 engagement and observed a dose-dependent reduction in the steady-state concentration of F2,6BP (Figure 2A). Whereas anti-CD3/anti-CD28-induced F2,6BP was suppressed by as little as 1 μM 3PO after five hours, 2-[1-^14^ C]-deoxy-D-glucose uptake stimulated by CD3/CD28 engagement was inhibited by 1 μM 3PO only after 24 hours (Figure 2B). Lactate secretion was markedly increased early during T cell activation and as little as 1 μM 3PO suppressed lactate secretion after only 10 hours (Figure 2C). The intracellular ATP concentration, which was not increased by the anti-CD3/anti-CD28-conjugated microbeads, also was inhibited but only after 24 hours of exposure to 5–10 μM 3PO (Figure 2D). These data support the conclusion that 3PO inhibits anti-CD3/anti-CD28-induced F2,6BP synthesis which in turn results in reduced glucose uptake and a subsequent decrease in ATP concentration. Direct visualization of the cells by light microscopy identified an early (10 hours) and substantial reduction in anti-CD3/anti-CD28-induced aggregation with exposure to as little as 1 μM of 3PO (Figure 2E). We next examined the effect of the anti-CD3/anti-CD28-conjugated microbeads on the secretion of TNF-α by the T cells as an early indicator of T cell activation [24]. Using an ELISA, we found that TNF-α secretion was markedly increased within 5 hours of T cell activation and that as little as 5 μM of 3PO reduced the increase in TNF-α secretion (Figure 2F). These data suggest that the observed reduction in the stimulation of F2,6BP and glucose uptake caused by 3PO may be sufficient to disrupt the synthesis of anabolic precursors and ATP necessary for the production and secretion of inflammatory mediators unrelated to metabolism.

### The PFKFB3 inhibitor 3PO increases T cell death and suppresses T cell proliferation after activation by anti-CD3/anti-CD28-conjugated microbeads

**Figure 3:**
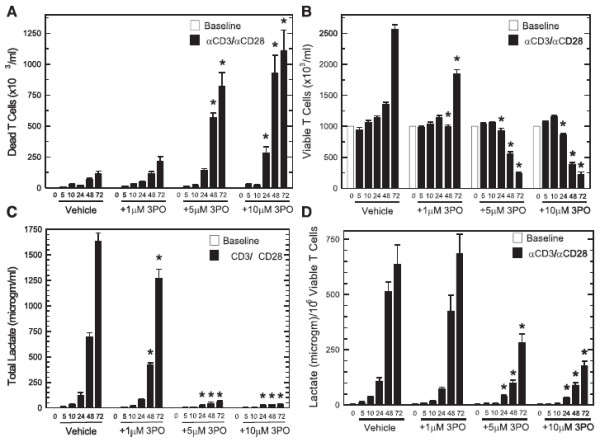
**A PFKFB3 small molecule antagonist causes cell death and a reduction of proliferation caused by anti-CD3/anti-CD28-conjugated microbeads after 24–72 hours.** Either vehicle (DMSO) or 1–10 μM 3PO was added to human CD3^+^ T cells stimulated with anti-CD3/anti-CD28-conjugated microbeads and IL-2 for 5, 10, 24, 48 and 72 hours and dead (**A**) and viable (**B**) T cells were enumerated with a New Brunswick Nucleocounter based propidium iodide staining with and without lysis. (**C**), Lactate secretion after addition of anti-CD3/anti-CD28-conjugated microbeads in the presence or absence of the indicated concentrations of 3PO for 5–72 hours. (**D**), Lactate secretion after addition of anti-CD3/anti-CD28-conjugated microbeads in the presence or absence of the indicated concentrations of 3PO for 5–72 hours was normalized to viable T cell counts. Data are representative of three independent experiments. *p < 0.05 (relative to vehicle control).

In order to examine the effects of 3PO on the viability and proliferation of T cells, we cultured CD3^+^ T cells with both anti-CD3/anti-CD28-conjugated microbeads and IL-2 in the presence of vehicle or 1–10 μM 3PO. We analyzed viable versus dead T cells at multiple time points using a New Brunswick NucleoCounter. The viable T cell counts were determined as the difference between the number of unlysed T cells that were detected after staining with propidium iodide (*i.e.* membrane-permeable, dead cells) and the number of T cells that were detected after lysis (*i.e.* total cells). As demonstrated in Figures 3A and 3B, cell death and a reduction in cell proliferation only occur as early as 24 hours after exposure to 10 μM and 5 μM 3PO, respectively. In contrast, 5–10 μM 3PO suppresses lactate and TNF-α secretion within ten hours of activation (Figure 2C and 2F). Although lactate secretion and cell proliferation are widely considered to be tightly linked given the anabolic precursors that are supplied by glycolysis and necessary for proliferation, including the ribose moiety of nucleic acids and several amino acids, we still observed a reduction of lactate secretion *per viable T cell* 24–72 hours after T cell activation (Figure 3C and 3D). The observation that the anti-metabolic effects of 3PO (Figures 2A, 2B and 2C) occur at an earlier time (*i.e.* 10 hrs) than the cytotoxic (Figure 3A; 24 hrs) and anti-proliferative effects (Figure 3B; 24 hrs) provides support for the conclusion that 3PO disrupts glycolytic metabolism which, in turn, results in a reduction of cell expansion and an increase in cell death.

### Suppression of the stimulation of DTH by i.p. administration of 3PO

**Figure 4:**
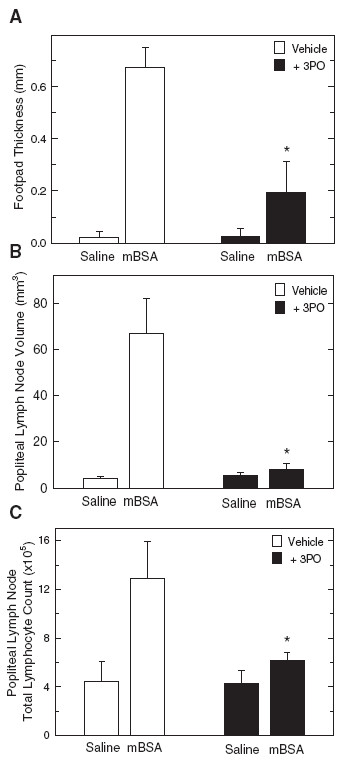
**Intraperitoneal administration of 3PO suppresses delayed type hypersensitivity to mBSA.** (**A**), Six BALB/c mice were injected subcutaneously with 250 μg mBSA emulsified with CFA and, seven days following immunization, the mice were challenged with an injection of 50 μl of 0.5 mg/ml mBSA in saline in one rear foot pad and 50 μl saline in the other rear footpad and then administered either vehicle (DMSO) or 3PO (0.07 mg/gm) 30 minutes and 12 hrs after the challenge. Footpad thickness was measured 24 hours following mBSA challenge using microcalipers. After the mice were euthanized, the draining popliteal lymph nodes were removed and measured with microcalipers (**B**) and the total number of lymphocytes counted using light microscopy (**C**). Data are representative of three independent experiments. *p < 0.05 (relative to vehicle control).

Given the relative potency of 3PO against T cell activation *in vitro*, we postulated that administration of this agent might attenuate T cell activation *in vivo*. Using an established, T cell-dependent model of DTH [23,25], we vaccinated BALB/c mice with methylated BSA (mBSA) emulsified in CFA. After 7 days, the mice were challenged with mBSA or control saline injection into a rear footpad and then, after 30 minutes and 12 hours, were administered either vehicle (DMSO) or 3PO (0.07 mg/gm). mBSA injection increased footpad thickness by approximately 600 μm after 24 hours in the vehicle-treated mice (Figure 4A). Intraperitoneal administration of 3PO markedly attenuated this increase in footpad thickness and also reduced the size of the draining popliteal lymph nodes (Figure 4B) and the total lymphocytes present in these lymph nodes (Figure 4C). These data provide support for the potential utility of 3PO as a T cell immunosuppressive agent.

### 3PO suppresses the development of psoriatic skin scaling, thickening, epidermal hyperplasia and T cell infiltration

**Figure 5:**
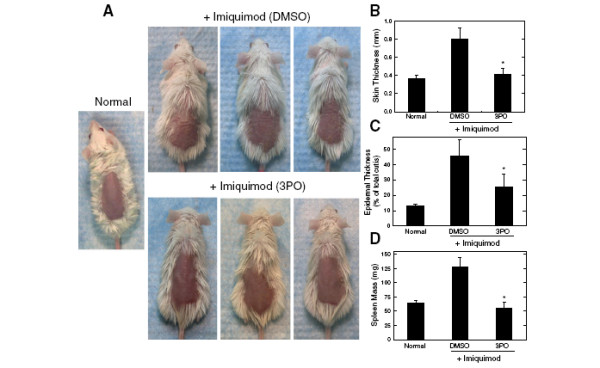
**Intraperitoneal administration of 3PO suppresses the development of imiquimod-induced psoriasis.** (**A**) Three BALB/c mice were administered a daily topical dose of 62.5 mg of imiquimod cream (5%) over a shaved area on the back for 5 days. Thirty minutes following each daily administration of imiquimod, the mice were injected intraperitoneally with either DMSO or 3PO 0.07 mg/gm daily. After 5 days, photographs were taken that demonstrate gross skin folds (*i.e.* wrinkling), scaling and erythema. A photograph was captured of a shaved mouse that had not been treated with imiquimod (Normal). (**B**), After 5 daily topical applications of imiquimod, skin thickness was measured using microcalipers. (**C**) After 5 daily topical applications of imiquimod, the epidermal thickness relative to the total cutis was determined. (**D**) After 5 daily topical applications of imiquimod, spleens were resected and mass was determined. Data from nine mice were averaged from three independent experiments (n = 3 per experiment). *p < 0.05 (relative to vehicle control). Data are representative of three independent experiments.

**Figure 6:**
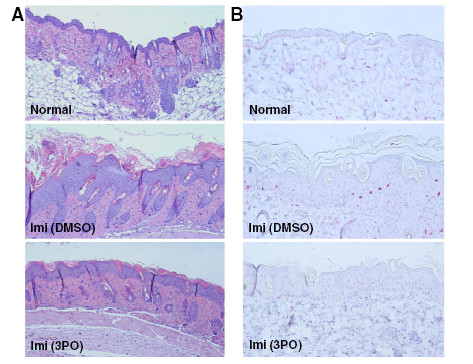
**3PO suppresses the increase in epidermal thickness and CD3**^**+**^**T cell infiltration caused by imiquimod.** BALB/c mice were administered a daily topical dose of 62.5 mg of imiquimod cream (5%) over a shaved area on the back for 5 days and thirty minutes following each daily administration of imiquimod, the mice were injected intraperitoneally with either DMSO or 3PO 0.07 mg/gm daily. (**A**) After 5 daily topical applications of imiquimod with intraperitoneal administration of DMSO or 3PO, the exposed skin was resected, fixed in formalin, embedded in paraffin, sectioned and stained with hematoxylin and eosin. (**B**) After 5 daily topical applications of imiquimod with intraperitoneal administration of DMSO or 3PO, the exposed skin was resected, fixed in formalin, embedded in paraffin, sectioned and CD3^+^ T cells were detected by immunohistochemistry. Data are representative of three independent experiments.

The toll-like receptor 7 ligand, imiquimod, has clinical activity against warts and precancerous skin lesions including actinic keratoses and basal cell carcinomas but has been found to exacerbate psoriasis in humans [26]. Similarly, application of imiquimod onto the shaved skin of BALB/c mice causes a psoriasis-like constellation of histopathological and clinical signs including epidermal hyperplasia, infiltration of T cells, neutrophils and dendritic cells, and thickening with plaque-associated scales and erythema [27]. Based on these observations, topical application of imiquimod onto mouse skin has been established as a valid model of psoriasis [26]. Human psoriasis is a T cell-dependent autoimmune disorder and depletion of T cells using anti-CD3 antibodies reduces the severity of imiquimod-induced psoriasis in mice [27]. Given that 3PO is a potent suppressor of T cell activation *in vitro*, we postulated that this agent would attenuate the progression of psoriasis caused by imiquimod. We applied imiquimod for a total of five days and either injected vehicle (DMSO) or 3PO (0.07 mg/gm) intraperitoneally 30 minutes after the application. Administration of 3PO caused a marked decrease in pathologically enlarged skin folds and scaling caused by imiquimod but no change in the development of erythema (Figure 5A). Imiquimod causes clinical and histological increases in skin thickness, epidermal thickness and splenomegaly [27] and we found that i.p. administration of 3PO markedly attenuated the development of all three of these pathologies (Figure 5B-D). Histopathological analyses of the skin revealed that imiquimod-induced psoriasis was associated with epidermal hyperplasia and microscopic scaling (Figure 6A) as well as increased CD3^+^ T cell infiltration into the skin, both of which were reduced significantly by the administration of 3PO (compare Imi [DMSO] to Imi [3PO], Figures 6A and 6B). Taken together, these data indicate that inhibition of PFKFB3 attenuates the development of an established mouse model of psoriasis.

## Discussion

We have demonstrated that PFKFB3 expression is increased by engagement of CD3 and CD28 on human peripheral blood T cells and that a PFKFB3 small molecule antagonist, 3PO, suppresses the anti-CD3/anti-CD28-mediated stimulation of F2,6BP synthesis and glucose uptake. Importantly, we also found that the early anti-metabolic effects and suppression of aggregation and TNF-α secretion by 3PO occurred within 5–10 hours of activation whereas a modest increase in cell death occurred only after 24 hours of 3PO exposure. Lastly, we observed that i.p. administration of a non-toxic dose of 3PO attenuated the DTH to a T cell antigen *in vivo* and prevented the development of imiquimod-induced, T cell-dependent psoriasis in mice. Taken together, our observations indicate that competitive inhibition of PFKFB3 kinase activity can attenuate the metabolism of activated T cells *in vitro* and cause suppression of immunity *in vivo.*

The finding that signaling through the TCR and CD28 increased PFKFB3 (but not PFKFB1, 2 or 4) mRNA and protein expression simultaneously with a >20-fold increase in its product, F2,6BP, suggests that PFKFB3 may be the dominant PFKFB family member involved in TCR/CD28-induced F2,6BP synthesis and glycolysis. However, we also observed that TIGAR protein expression decreased 5 hours after T cell activation, which could contribute to the early increase in F2,6BP, but then increased along with PFKFB3 expression after 10 and 24 hours. The rationale for such a coupled increase in the expression of a kinase and bisphosphatase that have opposing effects on the intracellular concentration of F2,6BP is not immediately apparent but may reflect a cellular response to maintain an optimal, steady state concentration of F2,6BP for cell activation. Alternatively, PFKFB3 and/or TIGAR may have additional functions required during T cell activation. At a minimum, these expression data indicate that the kinase domain of the PFKFB3 family member is an attractive regulatory enzyme target for the development of immunosuppressive agents.

Small molecule antagonists of kinases as well as si/shRNA molecules have been well established to have off-target effects and the immunosuppressive effects of 3PO conceivably could be unrelated to its inhibitory effects on PFKFB3 activity. Importantly, while the *K*_*m*_ of PFKFB3 for fructose-6-phosphate (F6P) is 97 μM, the *K*_*i*_ for 3PO competitive inhibition is 25±9 μM and 3PO has no effect on the activity of purified PFK-1 which shares the identical substrate, F6P (21)**.** Additionally, 3PO causes a reduction in F2,6BP, glucose uptake and lactate secretion by Jurkat T cell leukemia cells that precedes its cytostatic and cytotoxic effects (21). Last, ectopic expression of PFKFB3 increases intracellular F2,6BP and protects Jurkat T cell leukemia cells from the cytostatic effects of 3PO whereas heterozygous PFKFB3^+/−^ LT/*ras*-transformed fibroblasts that express decreased PFKFB3 protein and low F2,6BP compared to their wild-type isogenic counterparts have been found to be more sensitive to 3PO (21). Although these prior observations substantially support the conclusion that the suppression of intracellular PFKFB3 activity is the main mechanism of action that causes the cytostatic/toxic effects of 3PO, we acknowledge that unidentified off-target effects could, in part, cause the immunosuppressive effects of 3PO.

A second generation 3PO derivative (PFK015) was recently reported to more potently inhibit recombinant human PFKFB3 activity, F2,6BP synthesis and proliferation in cancer cells and to display markedly improved pharmacokinetic properties (2011 American Association for Cancer Research Annual Meeting, Abstract #2825) and a related compound is proceeding into phase I clinical testing in cancer patients. We suspect that these clinical grade 3PO derivatives may have utility in psoriasis as well as a wide spectrum of autoimmune and inflammatory disorders, such as rheumatoid arthritis, multiple sclerosis, systemic lupus erythematosus, inflammatory bowel disease, scleroderma, graft-versus-host disease and transplanted organ rejection. Glucocorticoids (*e.g.* prednisone) are now widely used for the management of these autoimmune and inflammatory disorders but are limited by serious side effects including osteonecrosis, hypertension, myopathy, and cataracts. Although clinical-grade PFKFB3 inhibitors have not yet undergone phase I testing, the parental compound 3PO did not cause toxicity in mice [21] and, in the current study, displayed significant immunosuppressive activity against two T cell-dependent models of immunity *in vivo*. Given the results of the current study, the more potent 3PO derivatives may cause immunosuppression that could put cancer patients at increased risk for opportunistic infections. This concern will be mitigated by close safety monitoring during the anticipated trials in cancer patients.

Whereas this study provides significant support for targeting PFKFB3 as a T cell activation inhibitor, it is possible that additional inflammatory cells may be affected by PFKFB3 small molecule antagonists. Macrophages, dendritic cells and B cells are each suspected to contribute to the development of DTH and to the pathogenesis of psoriasis, and their activation may be suppressed by agents that inhibit PFKFB3 and glycolysis. Additionally, each of these immune cells can function as antigen presenting cells and the *in vivo* effects of 3PO that we have observed may be in part related to a dysfunction in antigen presentation to T cells. Conditional PFKFB3 knock-out mice in which the expression of Cre is controlled by transcription factors that are immune lineage-specific should enable a complete characterization of the requirement of PFKFB3 expression for T cell activation as well as innate immune responses. On-going studies utilizing these mice are directed at understanding the role of this family of metabolic regulators in the activation of multiple lineages of immune cells and the anti-inflammatory effects of cell type-specific inhibition of PFKFB3.

## Conclusions

**Figure 7:**
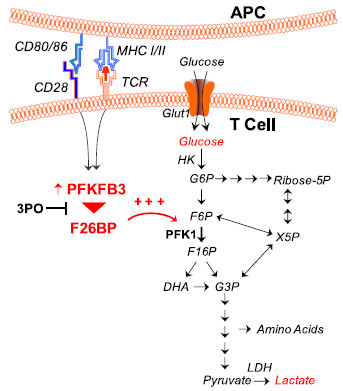
**3PO suppresses the CD3/CD28-induced activation of glycolysis in T cells.** Activation of T cells by anti-CD3/anti-CD28-conjugated microbeads causes a marked increase in PFKFB3 expression, F2,6BP, glucose uptake and lactate secretion. The PFKFB3 antagonist, 3PO, blocks T cell activation-induced glycolysis which, in turn, results in suppression of T cell function.

In these studies, we have demonstrated that PFKFB3 expression and F26BP synthesis are increased by stimulation of human peripheral blood T cells and that a PFKFB3 small molecule antagonist, 3PO, suppresses the stimulation of F2,6BP synthesis and glucose uptake with a subsequent decrease in T cell activation (Figure 7). We also have shown that *in vivo* administration of 3PO attenuates the development of a T cell-dependent DTH response and imiquimod-induced T cell dependent psoriasis. These data indicate that inhibition of the PFKFB3 kinase activity can reduce the activation of T cells *in vitro* and cause suppression of immunity *in vivo* and that small molecule PFKFB3 antagonists may prove to be effective immunosuppressive agents.

## Abbreviations

PFKFB: 6-Phosphofructo-2-kinase/fructose-2,6-bisphosphatase; TIGAR: P53-inducible regulator of glycolysis and apoptosis; F2,6BP: Fructose-2,6-bisphosphate; 3PO: 3-(3-pyridinyl)-1-(4-pyridinyl)-2-propen-1-one.

## Competing interests

The authors have no competing interests to declare.

## Authors’ contributions

All authors have read and approved the final manuscript. The specific contributions of each author are: ST did the majority of the experiments and analysis; BFC conducted glucose uptake and ATP measurements; ACK conducted the real time RT-PCR experiments; ALC conducted the proliferation experiments and F2,6BP measurements; JOT assisted with the 3PO studies; RB assisted with the study design and interpretation of the data; JC conceived, designed and directed the entire study, interpreted all data and wrote the manuscript.
